# Cetuximab enhanced the efficacy of chemotherapeutic agent in ABCB1/P-glycoprotein-overexpressing cancer cells

**DOI:** 10.18632/oncotarget.5813

**Published:** 2015-10-19

**Authors:** Fang Wang, Yifan Chen, Lihua Huang, Tao Liu, Yue Huang, Jianming Zhao, Xiaokun Wang, Ke Yang, Shaolin Ma, Liyan Huang, Kenneth Kin Wah To, Yong Gu, Liwu Fu

**Affiliations:** ^1^ Collaborative Innovation Center for Cancer Medicine, State Key Laboratory of Oncology in South China, Sun Yat-Sen University Cancer Center, Guangzhou, China; ^2^ Guangdong Esophageal Cancer Institute, Guangzhou, China; ^3^ School of Pharmacy, the Chinese University of Hong Kong, Hong Kong, China; ^4^ Department of Thoracic Surgery, the First Affiliated Hospital of Sun Yat-Sen University, Guangzhou, Guangdong Province, China

**Keywords:** cetuximab, EGFR, multidrug resistance, ABCB1/P-glycoprotein, chemotherapeutic agent

## Abstract

The overexpression of ATP-binding cassette (ABC) transporters is closely associated with the development of multidrug resistance (MDR) in certain types of cancer, which represents a formidable obstacle to the successful cancer chemotherapy. Here, we investigated that cetuximab, an EGFR monoclonal antibody, reversed the chemoresistance mediated by ABCB1, ABCG2 or ABCC1. Our results showed that cetuximab significantly enhanced the cytotoxicity of ABCB1 substrate agent in ABCB1-overexpressing MDR cells but had no effect in their parental drug sensitive cells and ABCC1, ABCG2 overexpressing cells. Furthermore, cetuximab markedly increased intracellular accumulation of doxorubicin (DOX) and rhodamine 123 (Rho 123) in ABCB1-overexpressing MDR cancer cells in a concentration-dependent manner. Cetuximab stimulated the ATPase activity but did not alter the expression level of ABCB1 or block phosphorylation of AKT and ERK. Interestingly, cetuximab decreased the cell membrane fluidity which was known to decrease the function of ABCB1. Our findings advocate further clinical investigation of combination chemotherapy of cetuximab and conventional chemotherapeutic drugs in ABCB1 overexpressing cancer patients.

## INTRODUCTION

MDR is a well characterized broad pattern of cross resistance to various structurally unrelated drugs after exposure to a single drug, which is a formidable barrier to the successful cancer chemotherapy. The MDR phenotype can lead to increase of drug efflux and inadequate induction of apoptosis. Up to now, the mechanism associated with efflux of drugs has focused on the overexpression of the superfamily of ABC transporters that function as active drug efflux pump leading to extrude a wide range of structurally and mechanistically diverse anticancer drugs against a concentration gradient, thereby resulting in chemotherapy failure [1]. To date, 49 different members of ABC transporter family have been identified in the human genome and are classified into seven subfamilies (A–G) based on sequence similarities [2]. Among them, ABCB1, ABCC1, and ABCG2 play major roles in the development of MDR in cancer cells [3]. Overexpression of ABCB1 on the surface of cancer cells is considered as the most common explanation of MDR. ABCB1 can pump out a wide spectrum of compounds including vinca alkaloids, epipodophyllotoxins, taxanes and some tyrosine kinase inhibitors (TKIs) [4, 5]. On the other hand, ABCB1 expression can be induced by drug-exposure and eventually develop to MDR. A lot of MDR inhibitors have been developed to reverse MDR, some of which are being evaluated in clinical trials for their potential circumvention of anticancer drug resistance [6–11]. Unfortunately, no transporter inhibitors have been put into use in the clinic because of insufficient efficacy, unacceptable toxicity or unpredictable pharmacokinetic interactions [12, 13].

As a lipid flippase, ABCB1 has complex interplay with cell membrane and play major function by located in the lipid rafts [14, 15]. The biological membrane regulates the physical properties of membrane and the functions of membrane proteins. In particular, the changes in the cholesterol and the sphingomyelin on the membrane can regulate ATP activity, drug binding and transportation by altering the fluidity of the cell membrane [14, 16]. Sinicrope and his colleagues had shown that alteration of membrane lipid fluidity of canalicular membrane vesicles modulated the ABCB1-mediated accumulation of MDR-type drugs [15]. But the expression of P-gp per se has little effect on membrane fluidity or membrane potential [17]. What's more, many reversal agents have proved to change the fluidity of membrane by interacting with the composition of membrane directly or indirectly. Therefore, the function of ABCB1 has innumerable links with the fluidity of membrane. Consequently, a logical strategy to overcome ABC transporters-mediated MDR is to develop inhibitors that could alter the fluidity of membrane.

EGFR signal pathways are involved in the control of cell survival, cell cycle, angiogenesis, migration, invasion and metastatic potential of cells. Cetuximab is a chimeric human-murine monoclonal antibody of EGFR, directly against EGFR, which binds to EGFR with an affinity that is approximately five to ten times higher than that of endogenous ligands. Cetuximab blocks binding of endogenous EGFR ligands resulting in inhibition of receptor function and induces EGFR internalization [18]. In addition, cetuximab is given to patients treated for metastatic colorectal cancer (CRC) in combination with irinotecan when irinotecan-based therapy had failed [19]. The safety and efficacy of cetuximab combine with irinotecan has been studied in patients with EGFR-overexpressing CRC and shown reliable efficacy in clinical trial [19, 20]. However, the mechanism of this combined therapy remains unknown. As irinotecan is an ABCB1 substrate, here we try to explore the effect of cetuximab on the reversal of MDR. According to a recent study, activation of the EGFR signaling pathways would up-regulate the ABC-transport protein expression and increase the survival of resistant cells [21]. We hypothesized that cetuximab could inhibit ABCB1 functionality through blocking EGFR signaling pathways. Indeed, many studies had also proved that the action of ABCB1 modulator appears to depend on the alteration of cell membrane fluidizer. These spur intensified effort to determine the role of cetuximab in the fluidity of cell membrane. In this study, we investigated the effect of cetuximab on the reversal of MDR induced by ABC transporters.

## RESULTS

### ABC transporter was examined in cell lines

**Figure 1:**
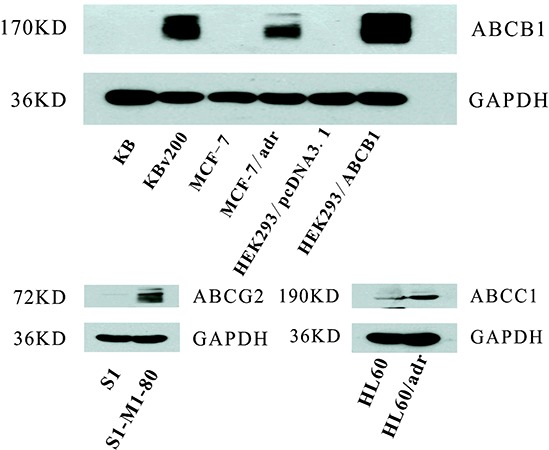
The ABC transpoter expression level The expression levels of ABCB1, ABCC1 and ABCG2 were measured by Western blot analysis as described in “Materials and Methods”. Western blot analysis confirmed that ABCB1 was overexpression in KBv200, MCF-7/adr and HEK293/ABCB1 cell lines, while undetectable expression of ABCB1 was observed in their parental sensitive KB, MCF-7, and HEK293/pcDNA3.1 cell lines. ABCC1 and ABCG2 were overexpression in HL-60/adr and S1-MI-80 cell lines, but not in their parental sensitive HL60 and S1 cell lines, respectively.

Western blot analysis confirmed that ABCB1 was overexpression in KBv200, MCF-7/adr and HEK293/ABCB1 cells, while undetectable expression levels were exhibited in their parental drug sensitive KB, MCF-7 and HEK293/pcDNA3.1 cells; and ABCC1 and ABCG2 were overexpression in HL60/adr and S1-MI-80 cells, but not in their parental sensitive HL60 and S1 cells, respectively (Figure 1).

### Cetuximab enhanced the cytotoxicity of conventional chemotherapeutic agent in ABCB1-overexpressing cells

We investigated the cytotoxicity of cetuximabin different cancer cells by MTT assay. Based on the concentration-effect curve, more than 90% of cells were viable under the concentrations of 250 μg/ml cetuximab in KBv200, MCF-7/adr and other cell lines. So we selected the concentration of ≤ 250 μg/ml cetuximab to reverse MDR. The cytotoxicity of ABCB1 substrate anticancer agent were detected in the presence or absence of cetuximab in KB and KBv200 cells, MCF-7 and MCF-7/adr cells, HEK293/pcDNA3.1 and HEK293/ABCB1 cells, respectively. Cetuximab exhibited a concentration-dependent decrease of the IC_50_ values of DOX and paclitaxel in KBv200, MCF-7/adr and HEK293/ABCB1 cells, but not increase the sensitivity in their parental sensitive KB, MCF-7 and HEK293/pcDNA3.1 cells, respectively (Table 1). On the other hand, cetuximab did not significantly enhance the cytotoxicity of non-ABCB1 substrate agent such as cisplatin, and also did not reverse ABCC1- or ABCG2-mediated MDR (Table 1). These results indicate that cetuximab is able to remarkably restore the sensitivity of ABCB1-overexpressing cells to ABCB1 substrate anticancer agent but has no effect on ABCC1-, ABCG2-mediated MDR.

**Table 1:** Effect of cetuximab on enhancing efficacy of chemotherapeutic agent

Compounds	IC50 ± SD(μM) (fold-resersal)	
	KB	KBv200(ABCB1)
Doxorubicin	0.0382 ± 0.0018 (1.00)	7.6657 ± 0.1981 (1.00)
+111.11 μg/ml Cetuximab	0.0372 ± 0.0008 (1.02)	3.7435 ± 0.2013 (2.05)**
+166.67 μg/ml Cetuximab	0.0407 ± 0.0049 (0.94)	2.5833 ± 0.6042 (2.96)**
+250 μg/ml Cetuximab	0.0369 ± 0.0041 (1.04)	1.1569 ± 0.5035 (6.63)**
+10 μM Verapamil	0.0418 ± 0.0026 (0.91)	0.1791 ± 0.0225 (42.80)**
Paclitaxel	0.0013 ± 0.0003 (1.00)	0.5749 ± 0.1651 (1.00)
+111.11 μg/ml Cetuximab	0.0012 ± 0.0002 (1.08)	0.2517 ± 0.0664 (2.28)*
+166.67 μg/ml Cetuximab	0.0009 ± 0.0001 (1.44)	0.1423 ± 0.0500 (4.04)*
+250 μg/ml Cetuximab	0.0016 ± 0.0002 (0.81)	0.0536 ± 0.0262 (10.73)**
+10 μM Verapamil	0.0009 ± 0.0001 (1.44)	0.0948 ± 0.0078 (6.06)**
Cisplatin	0.5504 ± 0.2003 (1.00)	0.8928 ± 0.1697 (1.00)
+250 μg/ml Cetuximab	0.5083 ± 0.0730 (1.08)	0.7710 ± 0.0597 (1.16)
	MCF-7	MCF-7/adr(ABCB1)
Doxorubicin	0.1278 ± 0.0525 (1.00)	1.7358 ± 0.1003 (1.00)
+111.11 μg/ml Cetuximab	0.1506 ± 0.0177 (0.85)	1.0596 ± 0.2713 (1.64)*
+166.67 μg/ml Cetuximab	0.1638 ± 0.0841 (0.78)	0.6542 ± 0.0940 (2.65)**
+250 μg/ml Cetuximab	0.0905 ± 0.0138 (1.41)	0.3184 ± 0.1155 (5.45)**
+10 μM Verapamil	0.0881 ± 0.0206 (1.45)	0.2150 ± 0.1175 (8.07)**
Cisplatin	6.9264 ± 0.4117 (1.00)	7.8635 ± 0.8816 (1.00)
+250 μg/ml Cetuximab	6.2955 ± 0.1742 (1.10)	8.3348 ± 0.2435 (0.94)
	HEK293/pcDNA3.1	HEK293/ABCB1
Doxorubicin	0.0320 ± 0.0057 (1.00)	0.6834 ± 0.0647 (1.00)
+111.11 μg/ml Cetuximab	0.0354 ± 0.0039 (0.90)	0.3649 ± 0.0534 (1.87)**
+166.67 μg/ml Cetuximab	0.0375 ± 0.0022 (0.85)	0.2333 ± 0.0522 (2.93)**
+250 μg/ml Cetuximab	0.0375 ± 0.0062 (0.85)	0.1861 ± 0.0197 (3.67)**
+10 uM Verapamil	0.0388 ± 0.0072 (0.82)	0.1068 ± 0.0192 (6.39)**
Cisplatin	2.8335 ± 0.4490 (0.97)	3.3188 ± 0.0089 (1.00)
+250 μg/ml Cetuximab	2.7511 ± 0.6060 (1.00)	3.0928 ± 0.3378(1.07)
	HL60	HL60/adr(ABCC1)
Doxorubicin	0.0243 ± 0.0079 (1.00)	3.7339 ± 0.0255 (1.00)
+111.11 μg/ml Cetuximab	0.0331 ± 0.0057 (0.73)	5.2110 ± 0.1010 (0.58)
+166.67 μg/ml Cetuximab	0.0260 ± 0.0028 (0.93)	4.9723 ± 0.6453 (0.61)
+250 μg/ml Cetuximab	0.0216 ± 0.0094 (1.13)	5.0766 ± 0.6372 (0.56)
+40 um MK571	0.0223 ± 0.0016 (1.09)	0.0900 ± 0.0417 (40.89)**
	S1	S1-MI-80(ABCG2)
Topotecan	0.3944 ± 0.0718(1.00)	22.6603 ± 0.0370(1.00)
+111.11 μg/ml Cetuximab	0.3823 ± 0.0963(1.03)	20.2467 ± 0.9366(1.12)
+166.67 μg/ml Cetuximab	0.3911 ± 0.0331(1.01)	21.1166 ± 0.0195(1.07)
+250 μg/ml Cetuximab	0.4884 ± 0.0694(0.81)	25.7293 ± 0.9608(0.88)
+2.5 uM FTC	0.3408 ± 0.0670(1.16)	4.7881 ± 0.1484(4.73)**

Cell viability was performed by MTT assay as described in “Materials and Methods”. Data was shown as the mean ± standard deviation (SD) of at least three independent experiments. The fold reversal of MDR (values given in parentheses) was calculated by dividing the IC_50_ value for cells with the anticancer drug in the absence of cetuximab by that obtained in the presence of cetuximab. Data represent Mean ± SD of at least three independent experiments.

“*” *P* < 0.05,

“**” *P* < 0.01.

### Cetuximab significantly increased the accumulation of DOX and Rho 123 in cells overexpressing ABCB1

**Figure 2:**
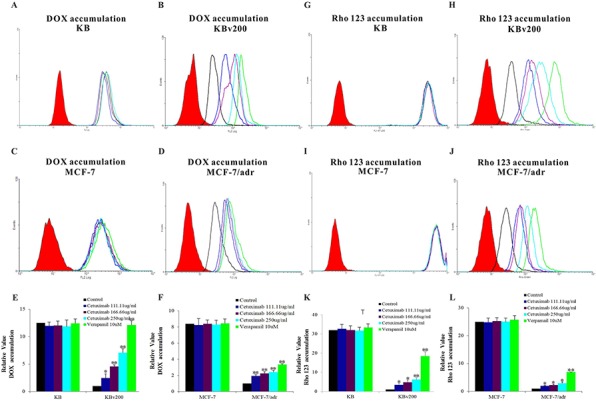
Effect of cetuximab on the accumulation of DOX and Rho 123 The accumulations of DOX **A, B, C, D.** and Rho 123 **G, H, I, J.** were measured by Flow cytometry analysis as described in “Materials and Methods”. The results **E, F, K, L.** were presented as fold change in fluorescence intensity relative to control MDR cells. Data represent Mean ± SD of at least three independent experiments. “*” *P* < 0.05, “**” *P* < 0.01.

It is well-known that the efflux of anticancer drug by ABCB1, leading to the reduction of intracellular drug accumulation and cell resistance. To investigate effect of cetuximab on the function of ABCB1, the intracellular accumulations of DOX and Rho 123 were examined in the presence or absence of cetuximab in ABCB1-overexpressing MDR cells and their parental drug sensitive cells. The intracellular accumulation of DOX or Rho 123 in KB and MCF-7 cells was higher than their resistant KBv200 and MCF-7/adr cells; and cetuximab significantly increased the accumulation of DOX and Rho 123 in KBv200 and MCF-7/adr cells in a concentration-dependent manner (Figure 2). In contrast, the cellular retention of DOX and Rho 123 were not altered in the parental sensitive cells in the presence of cetuximab (Figure 2). Taken together, these suggest that cetuximab inhibits the ABCB1 function of extrusion drug out of cells.

### ATPase activity of ABCB1 was stimulated by cetuximab

**Figure 3:**
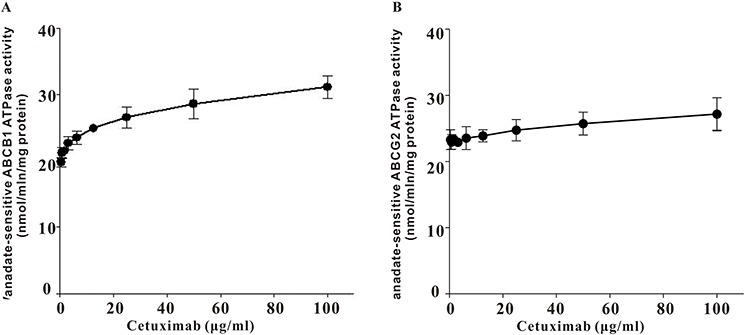
Effect of cetuximab on ATPase actvity of ABCB1 and ABCG2 Vanadate-sensitive ATPase activity of ABCB1 or ABCG2 was measured in the presence of different concentrations of cetuximab. Cetuximab significantly stimulated ABCB1 ATPase activity in a concentration-dependent manner but only slightly increase ABCG2 ATPase activiy. Data was shown as the Mean ± SD of at least three independent experiments.

Drug transport activities of ABCB1 and ABCG2 are associated with ATP hydrolysis that may be modulated by inhibitor of the transporter. To further understand the mechanisms of ABCB1 and ABCG2 function inhibition by cetuximab, vanadate-sensitive ATPase activities of both transporters were measured in the presence or absence of cetuximab (Figure 3). Cetuximab was found to stimulate ABCB1 ATPase activity in a concentration-dependent manner but have no obvious effect on the ABCG2 ATPase activity.

### Cetuximab did not significantly alter the expression of ABCB1 in protein or mRNA level

**Figure 4:**
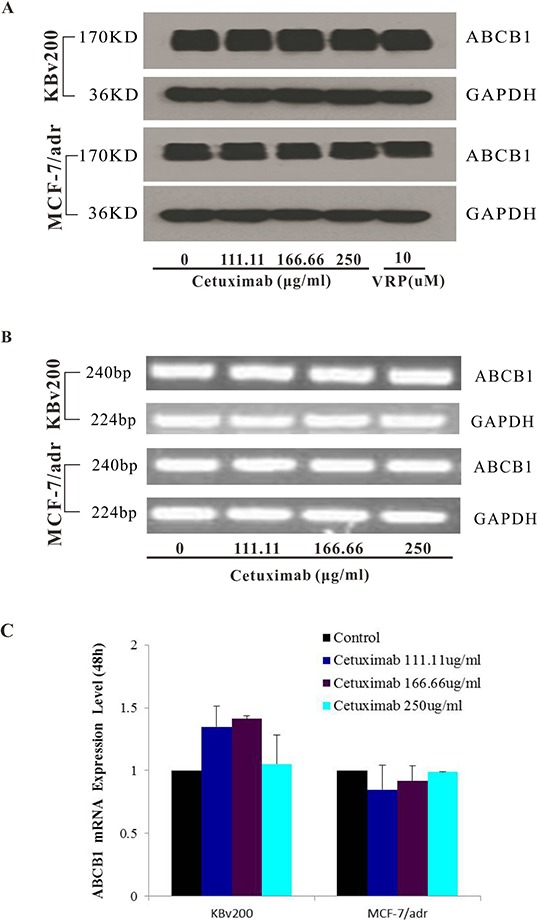
Effect of cetuximab on the expression of ABCB1 in MDR cells The protein level of ABCB1 was detected by Western blot analysis and mRNA level was measured by PCR/q-PCR analysis. Cetuximab did not alter the protein and mRNA expression levels in KBv200 and MCF-7/adr cells **A,B,C.** All experiments were repeated at least three times, and a representative experiment is shown in each panel. The 2^−ΔΔCt^ method wasused to analyze the relative change. Data represent Mean ± SD of at least three independent experiments. “*” *P* < 0.05, “**” *P* < 0.01.

The inhibition of ABC transporter function could be achieved by down-regulate the expression level of ABC transporter. Hence, we explored the effects of cetuximab on ABCB1 expression levels in mRNA and protein. Our results showed that cetuximab did not significantly alter the mRNA or protein level of ABCB1 in KBv200 and MCF-7/adr cells (Figure 4). These results indicated that the reversal of ABCB1-mediated MDR did not involve in the inhibition of ABCB1 expression.

### Interaction between ABCB1 and EGFR was not observed by co-immunoprecipitation

**Figure 5:**
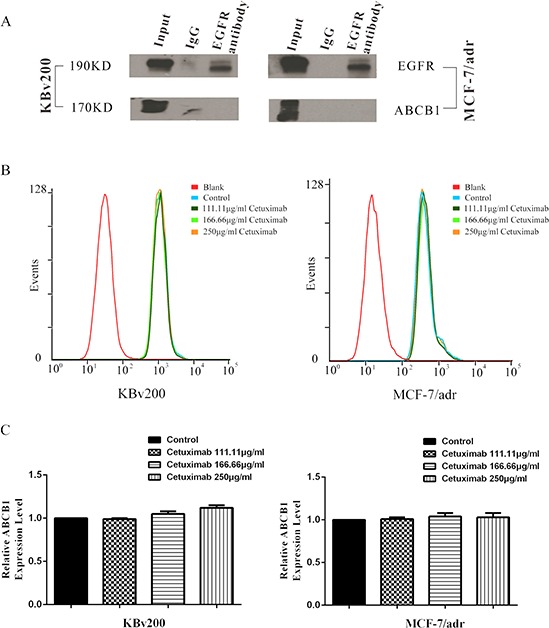
Effect of cetuximab on interaction between ABCB1 and EGFR The interaction between ABCB1 and EGFR was analysis by the co-immunoprecipitation assay as described in “Materials and Methods”. The result showed that there were no interaction between ABCB1 and EGFR **A.** The epimembranal ABCB1 protein was measured by the Flow cytometry as described in “Materials and Methods”. ABCB1 downregulation was not observed by Flow cytometry examination **B,C.**

In the previous study, cetuximab combines with EGFR can induce EGFR endocytosis and finally inhibit the function of EGFR signaling pathway. Here we hypothesis that cetuximab binding to EGFR may result in ABCB1 endocytosis after EGFR interact with ABCB1. Co-immunoprecipitation assay was used to detect the interaction between EGFR and ABCB1 after cetuximab treatment. The interaction between ABCB1 and EGFR was not observed in presence of cetuximab (Figure 5A). On the other hand, ABCB1 expression did not reduce in presence of cetuximab by Flow cytometry (Figure 5B, 5C).

### Cetuximab did not block the phosphorylation of AKT and ERK at MDR reversal concentrations

**Figure 6:**
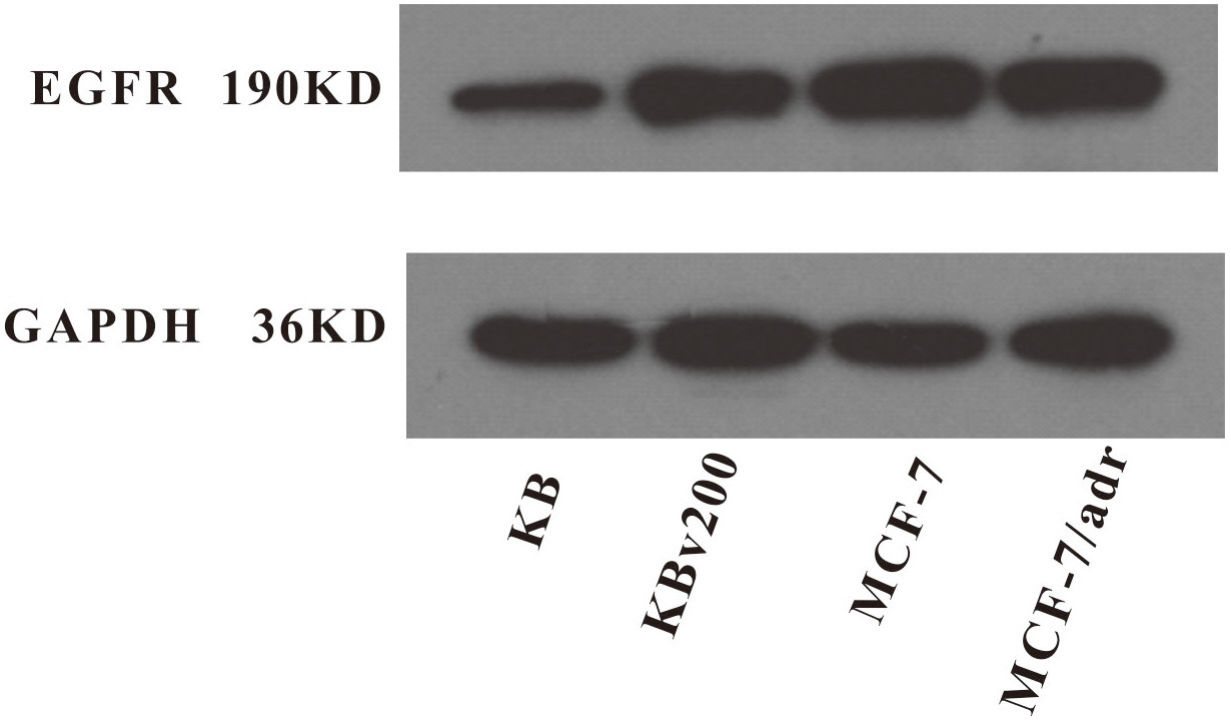
Expression level of EGFR in MDR cancer cells The protein level of EGFR was measured by western blot analysis as described in “Materials and Methods”. Western blot analysis confirmed that the EGFR expression level in KBv200 was higher than KB, but no difference between MCF-7/adr and MCF-7 cells.

**Figure 7:**
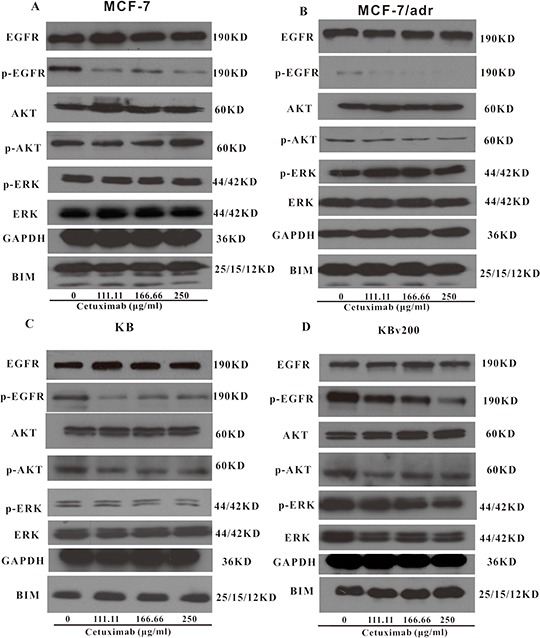
Effect of cetuximab on blockage of phosphorylation of EGFR, AKT and ERK The *p*-EGFR level was decreased after cetuximab treatment, but it did not alter the total or phosphorylated forms of AKT and ERK in KB, KBv200, MCF-7 and MCF-7/adr cells, respectively.

The activations of AKT and ERK pathways could increase the resistance to antineoplastic drugs in cancer cells [22]. To determine whether the cetuximab used in our experimental concentrations attenuated cell survival signaling pathways, we measured the change of total and phosphorylated forms of AKT and ERK in KB, KBv200, MCF-7 and MCF-7/adr cells. Firstly, Western blot confirmed that the EGFR expression level in KBv200 was higher than KB, but no difference between MCF-7/adr and MCF-7 cells (Figure 6). As shown in Figure 7, the p-EGFR level were slightly decreased after cetuximab treatment, but it did not alter the total or phosphorylated forms of AKT and ERK in KB, KBv200, MCF-7 and MCF-7/adr cells, respectively. These results suggest that MDR reversal effect of cetuximab is independent of the blockades of AKT and ERK signal transduction pathways in KBv200 and MCF-7/adr cells.

### Cetuximab decreased the fluidity of tumor cell membrane in MDR cells

**Figure 8:**
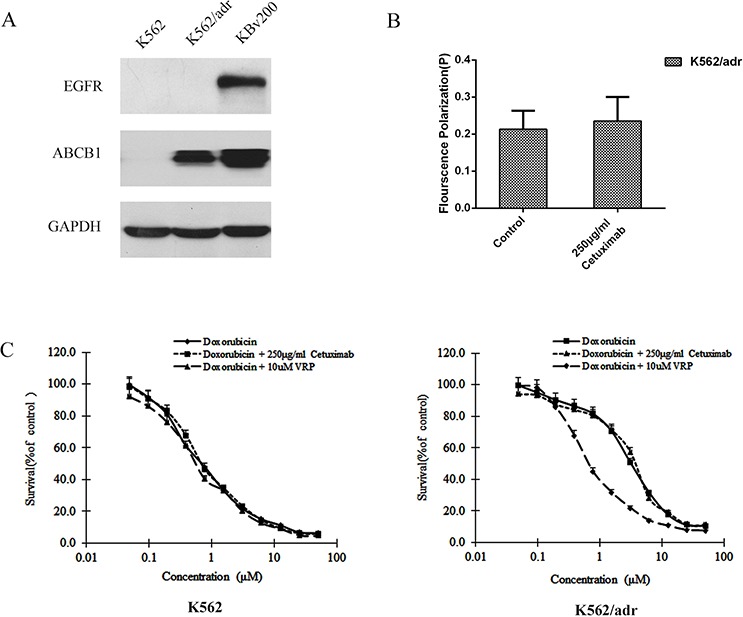
Effect of cetuximab on the cell membrane fluidity in EGFR negative K562/adr cells The protein level of EGFR was measured by western blot analysis as described in “Materials and Methods”. Western blot analysis confirmed that the EGFR was negative in K562 and K562/adr cells and positive in KBv200 cells **A.** A fluidity of tumor cell membrane assay was performed as described in “Materials and Methods”. Cetuximab did not alter the fluorescence polarization and fluidity of the membrane in K562/adr cells **B.** The MTT assay was performed as described in “Materials and Methods”. Cetuximab did not enhanced the cytotoxicity of DOX in ABCB1-overexpressing K562/adr cells but verapamil could do in K562/adr cells **C.**

The decrease of fluidity of tumor cell membrane resulted in the inhibition of ABCB1 function [15, 17]. We used 1,6-diphenyl-1,3,5-hexatriene (DPH), a kind of fluorescence probe, to mark the lipid located in the membrane and the fluorescence polarization to determent the fluidity of the membrane. Cetuximab was found to increase the fluorescence polarization and reduce the fluidity of the membrane in KBv200 and MCF-7/adr cells moderately in a concentration-dependent manner (Table 2). These results indicate that the inhibition of ABCB1 function and reversal of MDR by cetuximab are associated with the decrease the fluidity of membrane. Importantly, cetuximab did not alter the fluorescence polarization and fluidity of the membrane in the EGFR negative ABCB1 overexpressing K562/adr cells (Figure 8A, 8B). Moreover, cetuximab failed to enhance the cytotoxicity of DOX but verapamil could do in ABCB1-overexpressing K562/adr cells (Figure 8C). These indicate the reversal of ABCB1-mediated MDR by cetuximab depends on EGFR expression.

**Table 2:** Effect of cetuximab on fluidity of membrane in ABCB1 transporter overexpressing cells

	Flourscence Polarization(P)	
	KBv200	MCF-7/adr
Control	0.285 ± 0.007	0.229 ± 0.003
111.11 μg/ml Cetuximab	0.297 ± 0.010*	0.241 ± 0.005*
166.66 μg/ml Cetuximab	0.302 ± 0.004**	0.246 ± 0.013*
250 μg/ml Cetuximab	0.308 ± 0.008**	0.252 ± 0.003**

A fluidity of tumor cell membrane assay was performed as previously described in “Materials and Methods”. Data represent Mean ± SD of at least three independent experiments.

“*” *P* < 0.05,

“**” *P* < 0.01.

## DISCUSSION

Multidrug resistance of tumor cells is known to be the major barrier for successful cancer chemotherapy. Energy-dependent efflux of chemotherapeutic agent by ABC transporters, especially by ABCB1, ABCC1 and ABCG2, has been reported as important contributing factor to the development of MDR [24, 25]. Current strategies against MDR are to reverse MDR or prevent from MDR by MDR inhibitors. In the recent years, one potential finding is that the combination of cetuximab and irrnotecan is likely to have an additional beneficial effect in colorectal cancers. Indeed, the safety and efficacy of cetuximab combine with irinotecan has been studied in patients with EGFR-overexpressing CRC, and have shown reliable efficacy in clinic [19]. However, the mechanism of this combined therapy remains unknown. As we know irinotecan is an ABCB1 substrate, we hypothesized that cetuximab could effectively compete with chemotherapeutic agent binding to ABCB1 and thus increase intracellular drug accumulation in resistant cancer cells.

We found that cetuximab could increase the sensitivity of ABCB1-overexpressing cells to substrate chemotherapeutic agent in a concentration-dependent manner, but did not potentiate the cytotoxicity of non-substrate chemotherapeutic agent such as cisplatin. Importantly, cetuximab did not enhance the efficacy of chemotherapeutic agent in their parental drug sensitive cells. On the other hand, cetuximab couldn't increase the cytotoxicity of topotecan in ABCG2 overexpressing S1-MI-80 cells and the cytotoxicity of DOX in ABCC1 overexpressing HL60/adr cells. These demonstrate cetuximab could reverse ABCB1-mediated MDR but not to ABCC1- and ABCG2-mediated MDR. The extrusion anticancer drug out of the cells and the decrease of intracellular accumulation of anticancer drug was linked to MDR occurring. Drug accumulation and efflux were measured by flow cytometry. We found that cetuximab inhibited the efflux capacity of ABCB1 and increased intracellular accumulation of chemotherapeutic agent in a concentration-dependent manner in MDR cells. Therefore, the ability of cetuximab to reverse ABCB1-mediated MDR may be explained by the inhibitory effect on the drug efflux function of ABCB1.

**Figure 9:**
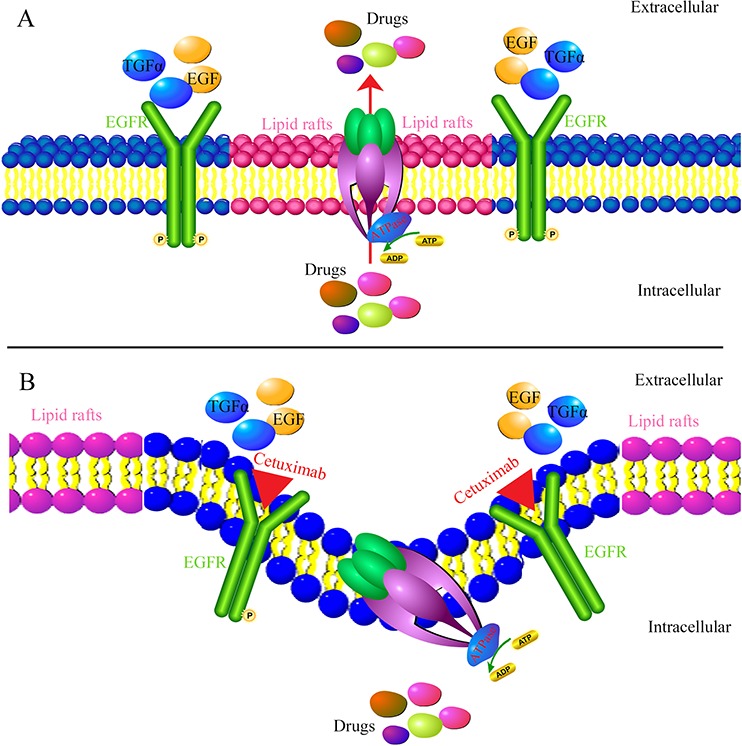
Mechanism of cetuximab reversing MDR When absence of cetuximab, EGF or TGFα can bind to the EGFR resulting in phosphorylation of EGFR and activation of EGFR signaling pathways. As a lipid flippase, ABCB1 has complex interplay with cell membrane and play major function by located in the lipid rafts **A.** However, at the presence of cetuximab, it can block the binding of endogenous EGFR ligands leading to the inhibition of receptor function and induce EGFR internalization. Cetuximab decrease cell membrane fluidity by regulating EGFR trafficking/turnover and facilitating a switch from lipid rafts to clathrin-mediated endocytosis. The change of the membrane fluidity inhibits the function of ABCB1. As a result, alteration of cell membrane fluidity inhibited the ABCB1 function of extrusion drug out of MDR cells **B.** Thus, cetuximab can enhance the efficacy of chemotherapeutic agent in ABCB1 mediated-MDR cells.

Extrusion of drug out of MDR cells by ABCB1 is dependent on energy support via ATP hydrolysis by ATPase [5]. So we detected the activities of ABCB1 and ABCG2 ATPases in the presence or absence of cetuximab. We found cetuximab significantly stimulated ABCB1 ATPase activity but only slightly increased ABCG2 ATPase activity. Some MDR inhibitors could down-regulate ABC transporter expression and reverse MDR [26]. To identify the effect of cetuximab on ABCB1 expression level, the mdr1 mRNA and ABCB1 protein were examined by q-PCR and Western blot, respectively. Cetuximab did not change the expression of ABCB1 at both the protein and mRNA levels. It had been reported that cetuximab could induce EGFR internalization [27]. Whether EGFR interacts with ABCB1 and induce its endocytosis in the presence of cetuximab? The co-immunoprecipitation assay showed that there is no interaction between EGFR and ABCB1 in the presence or absence of cetuximab. On the other hand, we did not observed ABCB1 downregulation by Flow cytometry examination (Figure 5B, 5C). This indicates that the reversal of MDR by cetuximab at the reverse concentrations do not link to ABCB1 endocytosis. Chen et al. reported that cetuximab decrease cell membrane fluidity by regulating EGFR trafficking/turnover and facilitating a switch from lipid rafts to clathrin-mediated endocytosis [28]. This suggests the decrease of cell membrane fluidity is dependent on the binding of cetuximab and EGFR. Furthermore, it was also reported that the alteration of cell membrane fluidity inhibited the ABCB1 function of extrusion drug out of MDR cells [29]. The change of the membrane fluidity inhibits the function of ABCB1 [30]. We found that the membrance fluidity was decreased after treated with cetuximab in MDR cells, which was linked to the inhibition of ABCB1 function. Interestingly, we found cetuximab could increase the fluorescence polarization and reduce the fluidity of the membrane in KBv200 and MCF-7/adr cells in a concentration-dependent manner. However, cetuximab did not alter the fluorescence polarization and fluidity of the membrane in the EGFR negative K562/adr cells (Figure 8A, 8B). Moreover, cetuximab failed to enhance the cytotoxicity of DOX in ABCB1-overexpressing K562/adr cells (Figure 8C). Thus, cetuximab probably depends on EGFR to decrease the cell membrane fluidity and further inhibit ABCB1 function (Figure 9).

In summary, cetuximab could enhance the cytotoxicity of conventional anticancer agent in ABCB1-overexpressing cancer cells by decreasing fluidity of MDR cell membrane and inhibiting the drug transport function and increasing intracellular accumulation of chemotherapeutic agent, but not involving in alteration of ABCB1 expression and the blockade of AKT and ERK signal transduction pathways in EGFR expression MDR cells. These findings encourage combinational chemotherapy of cetuximab and conventional anticancer drugs in EGFR expression MDR cancer patients.

## MATERIALS AND METHODS

### Chemicals and reagents

Cetuximab was purchased from Boehringer Ingelheim Pharma GmbH & Co. KG. Monoclonal antibodies against ABCB1, AKT, p-AKT, ERK, p-ERK and EGFR were purchased from Santa Cruz Biotechnology (CA, USA). Monoclonal antibody against p-EGFR and BIM were purchased from Cell Signaling Technology (Danvers, MA). Flow cytometry antibody against ABCB1 and IgG2α were purchased from BD Biosciences (San Jose, CA). Glyceraldehyde-3-phosphate dehydrogenase (GAPDH) antibody was purchased from Kangchen Co. (Shanghai, China). Dulbecco's modified Eagle's medium (DMEM) and RPMI 1640 medium were purchased from Life Technologies, Inc. Rho 123, 1-(4, 5-dimethylthiazol-2-yl)-3, 5-diphenylformazan (MTT), paclitaxel, DOX, verapamil (VRP), vincristine (VCR), topotecan, cisplatin, MK571, fumitremorgin C (FTC) and other chemicals were purchased from Sigma Chemical Co (St. Louis, MO).

### Cell lines and cell culture

The following cell lines were cultured in DMEM or RPMI 1640 supplemented with 10% FBS at 37°C in a humidified atmosphere of 5% CO_2_: the human oral epidermoid carcinoma cell line KB and its VCR-selected ABCB1-overexpressing derivative KBv200 [31]; the human breast carcinoma cell lines MCF-7 and its DOX-selected ABCB1-overexpressing derivative MCF-7/adr [32]; the human leukemia cell line K562 and its DOX-selected ABCB1-overexpressing derivative cell line K562/adr [33]; the human colon carcinoma cell lines S1 and its mitoxantrone (MX)-selected ABCG2-overexpressing derivative S1-MI-80 [34]; the human leukemia cell lines HL60 and its DOX-selected ABCC1-overexpressing derivative HL60/adr [35] and the human primary embryonic kidney cell line HEK293 and its pcDNA3.1, ABCB1 stable gene-transfected HEK293/pcDNA3.1 and HEK293/ABCB1 (cultured in medium with 2 mg/ml G418 [36]) cell lines, respectively. All cells were grown in drug free culture medium for more than 2 weeks before assay.

### Cell cytotoxicity assay

The MTT assay was performed as described previously to assess the sensitivity of cells to anticancer drugs [37]. The concentration required to inhibit cell growth by 50% (IC_50_) was calculated from survival curves using the Bliss method [38]. The degree of resistance was estimated by dividing the IC_50_ for the MDR cells by that of the parental sensitive cells; the fold-reversal factor of MDR was calculated by dividing the IC_50_ of the anticancer drug in the absence of cetuximab by that obtained in the presence of cetuximab.

### DOX and Rho 123 accumulation

The accumulations of DOX and Rho 123 were measured by flow cytometry as previously described [32]. Briefly, the cells were treated with cetuximab of various concentrations or vehicle at 37°C for 3 h. Then 10 μM DOX or 5 μM Rho 123 was added and incubation was continued for additional 3 h or 0.5 h, respectively. The cells were then collected, washed 3 times with ice-cold PBS, and analyzed by Flow cytometry analysis (Beckman Coulter, CytomicsFC500, USA). VRP, an ABCB1 inhibitor, was used as a positive control.

### ABCB1 and ABCG2 ATPase activity assay

A colorimetric ATPase assay was performed as previously described with minor modification [39]. Briefly, crude membranes isolated from High Five insect cells expressing either ABCB1 or ABCG2 (100 μg protein/mL) were incubated at 37°C with a range of different concentrations of cetuximab in the presence or absence of sodium orthovanadate (0.3 μM for ABCB1 and 1.2 μM for ABCG2) in ATPase assay buffer (50 mMKCl, 5 mM sodium azide, 2 mMEDTA, 10 mMMgCl2, 1 mMDTT, pH 6.8) for 5 min. The crude membranes were kind gift provided by Dr Suresh Ambudkar (National Cancer Institute, NIH, USA). ATP hydrolysis reaction was then started by the addition of 5 mM Mg-ATP (concentration in a final volume of 60 μL) and incubated for 20 min (for ABCB1) or 10 min (for ABCG2). SDS solution (30 μL of 10% SDS) was then added to terminate the reaction. After the addition of a detection reagent (35 mM ammonium molybdate, 15 mM zinc acetate, 10% ascorbic acid) and incubation at 37°C for 20 min, absorbance was measured at 750 nm. The amount of inorganic phosphate released was estimated by reading from a standard curve. Specific cetuximab-stimulated ABCB1 and ABCG2 ATPase activity (i.e. vanadate-sensitive) was determined as the difference between the amounts of inorganic phosphate released from ATP in the absence and presence of sodium orthovanadate.

### Western blot analysis

Western blot analysis was performed following a modification of methods described earlier [40]. To determine whether cetuximab affects the expression of ABCB1, EGFR, AKT, ERK, the phosphorylation of EGFR, AKT and ERK, the cells were treated with a range of different concentrations of cetuximab for 48 h.

### Flow cytometry analysis

Flow cytometry analysis was performed as described previously [41]. Expression of ABCB1 in the cell lines KBv200 and MCF-7/adr were measured by Flow cytometry after treated with a range of different concentrations of cetuximab for 48 h. Single-cell suspensions were prepared and washed three times with chilled PBS (supplemented with 0.5% bovine serum albumin). Then, 10 μl of phycoerythrin-conjugated, mouse anti-human ABCB1 antibody was mixed with 25 μl of cells (4 × 10^6^ cells per ml). After incubation in the dark for 45 min at 4°C, the cells were washed twice with chilled PBS (supplemented with 0.5% bovine serum albumin) and were resuspended in 400 μl of PBS for Flow cytometry analysis. Isotype control samples were treated in an identical manner with phycoerythrin-conjugated mouse IgG2α for ABCB1.

### PCR and real-time quantitative PCR

ABCB1 expression in mRNA level was assayed as previously described [40]. After a series of concentrations and different time periods of cetuximab treatment, total cellular RNA was isolated by trizol reagent RNA extraction kit following the manufacturer's instruction (Molecular Research Center, USA). The first strand cDNA was synthesized by OligodT primers with reverse transcriptase (Promega Corp. Madison, WI). The PCR primers were 5^'^-CAGGCTTGCTGTAATTACCCA-3^'^(forward) and 5^'^-TCAAAGAAACAACGGTTCGG-3^'^(reverse) for ABCB1; 5^'^-GAGTCAAGGATTTGGTCGT-3^'^(forward) and 5^'^-GATCTCGCTCCTGGAAGATG-3^'^(reverse) for GAPDH, respectively. After 35 cycles of amplification, products were resolved and examined by 1.0% agarose gel electrophoresis.

Real-time PCR was performed with Real-time PCR Master Mix containing SYBR GREEN I and hotStartTaq DNA polymerase. Real-time detection of the emission intensity of SYBR GREEN bound to double-stranded DNAs was performed using the iCycler Instrument (Bio-Rad, Hercules, CA, USA). The level of ABCB1 mRNA was expressed as a ratio relative to the GAPDH mRNA in each sample. Relative quantification of ABCB1 was performed using the 2 ^−ΔΔCt^ method [42]. The results were obtained from three reactions in each sample and analyzed by the SPSS software (Version 11.0) (SPSS Inc., Chicago, IL, USA).

### Co-immunoprecipitation assay

The co-immunoprecipitation assay was previously described with minor modification [43]. After KBv200 and MCF-7/adr cells were treated with a range of different concentrations of cetuximab for 48 h. The cells were lysed in RIPA solution. The lysates were centrifuged at 13,000 rpm for 30 min at 4°C. The supernatants were collected and total protein amount was measured using the BCA method [44]. For co-immunoprecipitation, the supernatant of cell lysate corresponding to 1 mg of total protein was precleared by Protein G-agarose beads to minimize nonspecific binding. Then the precleared supernatant was divided into three parts, two of which were incubated with 2 μg of anti-EGFR antibody or anti-IgG antibody for 1 h at 4°C separately, followed by incubation with protein G-agarose beads overnight at 4°C. The bound proteins were washed thrice with lysis buffer and dissociated with the beads via boiling and centrifugation. The collected proteins were suspended in 1 × protein loading buffer, separated by SDS-PAGE and analyzed by Western blot using the primary antibody of anti-ABCB1.

### Fluidity of tumor cell membrane assay

A Fluidity of tumor cell membrane assay was performed as Sinicrope FA et.al. described with minor modification [45]. The cells in logarithmic growth phase were treated with cetuximab of various concentrations at 37°C for 24 h. Then the cells were then collected, washed 3 times with ice-cold PBS. With the number of cells thus obtained, a cell suspension with 10^7^/mL was made. A 2 mL of the 4 mL tumor cell suspension was taken and added to a test tube as the blank tube, while the test tube containing the remaining 2 mL of the red cell suspension was used for reference. A 2 mL of DPH probe solution was added to the reference tube, while the same amount of isotonic PBS buffer solution was added to the blank tube. Solution in the two tubes was mixed and incubated for 30 min under a temperature of 25°C and then centrifuged for 5 min at 1500 r/min. The remaining DPH probe solution was discarded, and the residue was rinsed twice with isotonic PBS buffer solution and then diluted into 4 mL of cell suspension with isotonic PBS buffer solution. Immediately after this, the fluorescence polarization of the residue was measured. Spectra Max M Seriel Multiscan Spectrum was used to measure the intensity P of fluorescence polarized light both when it was parallel to and when it was perpendicular to the direction of vibration of the excitation polarized light, at a fluorescence excitation wavelength of 362 nm, a radiation wavelength of 432 nm, and under a temperature of 25°C.

### Statistics

Results were shown as means ± SD. All experiments were repeated at least three times and the differences were determined by using the Student's *t*-test. The statistical significance was determined to be *P* < 0.05.

## Abbreviation

ABC: ATP-binding cassette
CRC: colorectal cancer
DPH: 1,6-diphenyl-1,3,5-hexatriene
ERK: extracellular signal-regulated kinase
DOX: doxorubicin
VRP: verapamil
GAPDH: glyceraldehyde-3-phosphate dehydrogenase
IC50: half maximal inhibitory concentration
mAb: monoclonal antibody
MDR: multidrug resistance
MTT: 1-(4, 5-dimethylthiazol-2-yl)-3, 5-diphenylformazan
Rho 123: rhodamine 123
TKIs: tyrosine kinase inhibitors
